# Supplementary material for: Dysembryogenetic Pathogenesis of Basal Cell Carcinoma: The Evidence to Date
Source: Int J Mol Sci. 2024 Aug 2;25(15):8452. doi: 10.3390/ijms25158452 (PMC11312899; doi:10.3390/ijms25158452)
Supplement: Supplementary file 1 [file ijms-25-08452-s001.zip › ijms-3051616-supplementary.pdf]

Supplementary materials

PubMed search strategy

Search 1: (basal cell carcinoma) AND (embryology) = 351 records

Search details

("carcinoma, basal cell"[MeSH Terms] OR ("carcinoma"[All Fields] AND "basal"[All Fields] AND "cell"[All Fields]) OR "basal cell carcinoma"[All Fields] OR ("basal"[All Fields] AND "cell"[All Fields] AND "carcinoma"[All Fields])) AND ("embryology"[MeSH Terms] OR "embryology"[All Fields] OR "embryologies"[All Fields] OR "embryology"[MeSH Subheading])

Translations

basal cell carcinoma: "carcinoma, basal cell"[MeSH Terms] OR ("carcinoma"[All Fields] AND "basal"[All Fields] AND "cell"[All Fields]) OR "basal cell carcinoma"[All Fields] OR ("basal"[All Fields] AND "cell"[All Fields] AND "carcinoma"[All Fields])

embryology: "embryology"[MeSH Terms] OR "embryology"[All Fields] OR "embryologies"[All Fields] OR "embryology"[Subheading]

Search 2: ((basal cell carcinoma) AND (embryology)) AND (pathogenesis) = 242 records

Search details

("carcinoma, basal cell"[MeSH Terms] OR ("carcinoma"[All Fields] AND "basal"[All Fields] AND "cell"[All Fields]) OR "basal cell carcinoma"[All Fields] OR ("basal"[All Fields] AND "cell"[All Fields] AND "carcinoma"[All Fields])) AND ("embryology"[MeSH Terms] OR "embryology"[All Fields] OR "embryologies"[All Fields] OR "embryology"[MeSH Subheading]) AND ("etiology"[MeSH Subheading] OR "etiology"[All Fields] OR "pathogenesis"[All Fields])

Translations

basal cell carcinoma: "carcinoma, basal cell"[MeSH Terms] OR ("carcinoma"[All Fields] AND "basal"[All Fields] AND "cell"[All Fields]) OR "basal cell carcinoma"[All Fields] OR ("basal"[All Fields] AND "cell"[All Fields] AND "carcinoma"[All Fields])

embryology: "embryology"[MeSH Terms] OR "embryology"[All Fields] OR "embryologies"[All Fields] OR "embryology"[Subheading]

pathogenesis: "etiology"[Subheading] OR "etiology"[All Fields] OR "pathogenesis"[All Fields]

Search 3: (basal cell carcinoma) AND (embryo) = 224 records.

Search details

("carcinoma, basal cell"[MeSH Terms] OR ("carcinoma"[All Fields] AND "basal"[All Fields] AND "cell"[All Fields]) OR "basal cell carcinoma"[All Fields] OR ("basal"[All Fields] AND "cell"[All Fields] AND "carcinoma"[All Fields])) AND ("embryo s"[All Fields] OR "embryoes"[All Fields] OR "embryonic structures"[MeSH Terms] OR ("embryonic"[All Fields] AND "structures"[All Fields]) OR "embryonic structures"[All Fields] OR "embryo"[All Fields] OR "embryos"[All Fields])

Translations

basal cell carcinoma: "carcinoma, basal cell"[MeSH Terms] OR ("carcinoma"[All Fields] AND "basal"[All Fields] AND "cell"[All Fields]) OR "basal cell carcinoma"[All Fields] OR ("basal"[All Fields] AND "cell"[All Fields] AND "carcinoma"[All Fields])

embryo: "embryo's"[All Fields] OR "embryoes"[All Fields] OR "embryonic structures"[MeSH Terms] OR ("embryonic"[All Fields] AND "structures"[All Fields]) OR "embryonic structures"[All Fields] OR "embryo"[All Fields] OR "embryos"[All Fields]

Search 4: ((embryogenesis) AND (pathogenesis)) AND (basal cell carcinoma) = 189 records.

Search details

("embryonic development"[MeSH Terms] OR ("embryonic"[All Fields] AND "development"[All Fields]) OR "embryonic development"[All Fields] OR "embryogenesis"[All Fields]) AND ("etiology"[MeSH Subheading] OR "etiology"[All Fields] OR "pathogenesis"[All Fields]) AND ("carcinoma, basal cell"[MeSH Terms] OR ("carcinoma"[All Fields] AND "basal"[All Fields] AND "cell"[All Fields]) OR "basal cell carcinoma"[All Fields] OR ("basal"[All Fields] AND "cell"[All Fields] AND "carcinoma"[All Fields]))

Translations

embryogenesis: "embryonic development"[MeSH Terms] OR ("embryonic"[All Fields] AND "development"[All Fields]) OR "embryonic development"[All Fields] OR "embryogenesis"[All Fields]

pathogenesis: "etiology"[Subheading] OR "etiology"[All Fields] OR "pathogenesis"[All Fields]

basal cell carcinoma: "carcinoma, basal cell"[MeSH Terms] OR ("carcinoma"[All Fields] AND "basal"[All Fields] AND "cell"[All Fields]) OR "basal cell carcinoma"[All Fields] OR ("basal"[All Fields] AND "cell"[All Fields] AND "carcinoma"[All Fields])

Search 5: ((pathogenesis) AND (basal cell carcinoma)) AND (embryo) = 156 records.

Search details

("etiology"[MeSH Subheading] OR "etiology"[All Fields] OR "pathogenesis"[All Fields]) AND ("carcinoma, basal cell"[MeSH Terms] OR ("carcinoma"[All Fields] AND "basal"[All Fields] AND "cell"[All Fields]) OR "basal cell carcinoma"[All Fields] OR ("basal"[All Fields] AND "cell"[All Fields] AND "carcinoma"[All Fields])) AND ("embryo s"[All Fields] OR "embryoes"[All Fields] OR "embryonic structures"[MeSH Terms] OR ("embryonic"[All Fields] AND "structures"[All Fields]) OR "embryonic structures"[All Fields] OR "embryo"[All Fields] OR "embryos"[All Fields])

Translations

pathogenesis: "etiology"[Subheading] OR "etiology"[All Fields] OR "pathogenesis"[All Fields]

basal cell carcinoma: "carcinoma, basal cell"[MeSH Terms] OR ("carcinoma"[All Fields] AND "basal"[All Fields] AND "cell"[All Fields]) OR "basal cell carcinoma"[All Fields] OR ("basal"[All Fields] AND "cell"[All Fields] AND "carcinoma"[All Fields])

embryo: "embryo's"[All Fields] OR "embryoes"[All Fields] OR "embryonic structures"[MeSH Terms] OR ("embryonic"[All Fields] AND "structures"[All Fields]) OR "embryonic structures"[All Fields] OR "embryo"[All Fields] OR "embryos"[All Fields]

Search 6: (((embryogenesis) AND (pathogenesis)) AND (embryo)) AND (basal cell carcinoma) = 52 records.

Search details

("embryonic development"[MeSH Terms] OR ("embryonic"[All Fields] AND "development"[All Fields]) OR "embryonic development"[All Fields] OR "embryogenesis"[All Fields]) AND ("etiology"[MeSH Subheading] OR

"etiology"[All Fields] OR "pathogenesis"[All Fields]) AND ("embryo s"[All Fields] OR "embryoes"[All Fields] OR "embryonic structures"[MeSH Terms] OR ("embryonic"[All Fields] AND "structures"[All Fields]) OR "embryonic structures"[All Fields] OR "embryo"[All Fields] OR "embryos"[All Fields]) AND ("carcinoma, basal cell"[MeSH Terms] OR ("carcinoma"[All Fields] AND "basal"[All Fields] AND "cell"[All Fields]) OR "basal cell carcinoma"[All Fields] OR ("basal"[All Fields] AND "cell"[All Fields] AND "carcinoma"[All Fields]))

Translations

embryogenesis: "embryonic development"[MeSH Terms] OR ("embryonic"[All Fields] AND "development"[All Fields]) OR "embryonic development"[All Fields] OR "embryogenesis"[All Fields]

pathogenesis: "etiology"[Subheading] OR "etiology"[All Fields] OR "pathogenesis"[All Fields]

embryo: "embryo's"[All Fields] OR "embryoes"[All Fields] OR "embryonic structures"[MeSH Terms] OR ("embryonic"[All Fields] AND "structures"[All Fields]) OR "embryonic structures"[All Fields] OR "embryo"[All Fields] OR "embryos"[All Fields]

basal cell carcinoma: "carcinoma, basal cell"[MeSH Terms] OR ("carcinoma"[All Fields] AND "basal"[All Fields] AND "cell"[All Fields]) OR "basal cell carcinoma"[All Fields] OR ("basal"[All Fields] AND "cell"[All Fields] AND "carcinoma"[All Fields])

Search 7: (((basal cell carcinoma) AND (embryology)) AND (embryogenesis)) AND (pathogenesis) = 47 records

Search details

("carcinoma, basal cell"[MeSH Terms] OR ("carcinoma"[All Fields] AND "basal"[All Fields] AND "cell"[All Fields]) OR "basal cell carcinoma"[All Fields] OR ("basal"[All Fields] AND "cell"[All Fields] AND "carcinoma"[All Fields])) AND ("embryology"[MeSH Terms] OR "embryology"[All Fields] OR "embryologies"[All Fields] OR "embryology"[MeSH Subheading]) AND ("embryonic development"[MeSH Terms] OR ("embryonic"[All Fields] AND "development"[All Fields]) OR "embryonic development"[All Fields] OR "embryogenesis"[All Fields]) AND ("etiology"[MeSH Subheading] OR "etiology"[All Fields] OR "pathogenesis"[All Fields])

Translations

basal cell carcinoma: "carcinoma, basal cell"[MeSH Terms] OR ("carcinoma"[All Fields] AND "basal"[All Fields] AND "cell"[All Fields]) OR "basal cell carcinoma"[All Fields] OR ("basal"[All Fields] AND "cell"[All Fields] AND "carcinoma"[All Fields])

embryology: "embryology"[MeSH Terms] OR "embryology"[All Fields] OR "embryologies"[All Fields] OR "embryology"[Subheading]

embryogenesis: "embryonic development"[MeSH Terms] OR ("embryonic"[All Fields] AND "development"[All Fields]) OR "embryonic development"[All Fields] OR "embryogenesis"[All Fields]

pathogenesis: "etiology"[Subheading] OR "etiology"[All Fields] OR "pathogenesis"[All Fields]

Search 8: ((((basal cell carcinoma) AND (embryology)) AND (embryogenesis)) AND (embryo)) AND (pathogenesis) = 27 records

Search details

("carcinoma, basal cell"[MeSH Terms] OR ("carcinoma"[All Fields] AND "basal"[All Fields] AND "cell"[All Fields]) OR "basal cell carcinoma"[All Fields] OR ("basal"[All Fields] AND "cell"[All Fields] AND "carcinoma"[All Fields])) AND ("embryology"[MeSH Terms] OR "embryology"[All Fields] OR "embryologies"[All Fields] OR "embryology"[MeSH Subheading]) AND

("embryonic development"[MeSH Terms] OR ("embryonic"[All Fields] AND "development"[All Fields]) OR "embryonic development"[All Fields] OR "embryogenesis"[All Fields]) AND ("embryo s"[All Fields] OR "embryoes"[All Fields] OR "embryonic structures"[MeSH Terms] OR ("embryonic"[All Fields] AND "structures"[All Fields]) OR "embryonic structures"[All Fields] OR "embryo"[All Fields] OR "embryos"[All Fields]) AND ("etiology"[MeSH Subheading] OR "etiology"[All Fields] OR "pathogenesis"[All Fields])

Translations

basal cell carcinoma: "carcinoma, basal cell"[MeSH Terms] OR ("carcinoma"[All Fields] AND "basal"[All Fields] AND "cell"[All Fields]) OR "basal cell carcinoma"[All Fields] OR ("basal"[All Fields] AND "cell"[All Fields] AND "carcinoma"[All Fields])

embryology: "embryology"[MeSH Terms] OR "embryology"[All Fields] OR "embryologies"[All Fields] OR "embryology"[Subheading]

embryogenesis: "embryonic development"[MeSH Terms] OR ("embryonic"[All Fields] AND "development"[All Fields]) OR "embryonic development"[All Fields] OR "embryogenesis"[All Fields]

embryo: "embryo's"[All Fields] OR "embryoes"[All Fields] OR "embryonic structures"[MeSH Terms] OR ("embryonic"[All Fields] AND "structures"[All Fields]) OR "embryonic structures"[All Fields] OR "embryo"[All Fields] OR "embryos"[All Fields]

pathogenesis: "etiology"[Subheading] OR "etiology"[All Fields] OR "pathogenesis"[All Fields]

Search 9: (basal cell carcinoma) AND (hedgehog pathway inhibitor) = 603 records

Search details

("carcinoma, basal cell"[MeSH Terms] OR ("carcinoma"[All Fields] AND "basal"[All Fields] AND "cell"[All Fields]) OR "basal cell carcinoma"[All Fields] OR ("basal"[All Fields] AND "cell"[All Fields] AND "carcinoma"[All Fields])) AND (("hedgehogs"[MeSH Terms] OR "hedgehogs"[All Fields] OR "hedgehog"[All Fields]) AND ("pathway"[All Fields] OR "pathway s"[All Fields] OR "pathways"[All Fields]) AND ("antagonists and inhibitors"[MeSH Subheading] OR ("antagonists"[All Fields] AND "inhibitors"[All Fields]) OR "antagonists and inhibitors"[All Fields] OR "inhibitors"[All Fields] OR "inhibitor"[All Fields] OR "inhibitor s"[All Fields]))

Translations

basal cell carcinoma: "carcinoma, basal cell"[MeSH Terms] OR ("carcinoma"[All Fields] AND "basal"[All Fields] AND "cell"[All Fields]) OR "basal cell carcinoma"[All Fields] OR ("basal"[All Fields] AND "cell"[All Fields] AND "carcinoma"[All Fields])

hedgehog: "hedgehogs"[MeSH Terms] OR "hedgehogs"[All Fields] OR "hedgehog"[All Fields]

pathway: "pathway"[All Fields] OR "pathway's"[All Fields] OR "pathways"[All Fields]

inhibitor: "antagonists and inhibitors"[Subheading] OR ("antagonists"[All Fields] AND "inhibitors"[All Fields]) OR "antagonists and inhibitors"[All Fields] OR "inhibitors"[All Fields] OR "inhibitor"[All Fields] OR "inhibitor's"[All Fields]
